# A Smart Imaging Workflow for Organ-Specific Screening in a Cystic Kidney Zebrafish Disease Model

**DOI:** 10.3390/ijms20061290

**Published:** 2019-03-14

**Authors:** Gunjan Pandey, Jens H. Westhoff, Franz Schaefer, Jochen Gehrig

**Affiliations:** 1 Acquifer is a division of Ditabis, Digital Biomedical Imaging Systems AG, 75179 Pforzheim, Germany; gunjan.pandey@med.uni-heidelberg.de; 2 Department of Pediatrics I, University Children’s Hospital Heidelberg, 69120 Heidelberg, Germany

**Keywords:** zebrafish, high-content screening, automated imaging, image analysis

## Abstract

The zebrafish is being increasingly used in biomedical research and drug discovery to conduct large-scale compound screening. However, there is a lack of accessible methodologies to enable automated imaging and scoring of tissue-specific phenotypes at enhanced resolution. Here, we present the development of an automated imaging pipeline to identify chemical modifiers of glomerular cyst formation in a zebrafish model for human cystic kidney disease. Morpholino-mediated knockdown of intraflagellar transport protein Ift17*2* in *Tg(wt1b:EGFP)* embryos was used to induce large glomerular cysts representing a robustly scorable phenotypic readout. Compound-treated embryos were consistently aligned within the cavities of agarose-filled microplates. By interfacing feature detection algorithms with automated microscopy, a smart imaging workflow for detection, centring and zooming in on regions of interests was established, which enabled the automated capturing of standardised higher resolution datasets of pronephric areas. High-content screening datasets were processed and analysed using custom-developed heuristic algorithms implemented in common open-source image analysis software. The workflow enables highly efficient profiling of entire compound libraries and scoring of kidney-specific morphological phenotypes in thousands of zebrafish embryos. The demonstrated toolset covers all the aspects of a complex whole organism screening assay and can be adapted to other organs, specimens or applications.

## 1. Introduction

Besides its role as a classic model in developmental biology, the zebrafish embryo is being increasingly used in biomedical research to decipher disease-associated biological processes, with a high potential for subsequent drug discovery applications [1,2,3]. The availability of thousands of transgenic and mutant lines [4] provides a compelling resource for visualising and analysing healthy and pathological biological processes in the context of a live vertebrate animal. This is complemented by a vast toolbox of genetic and biochemical techniques allowing for rapid modelling of various human disease phenotypes, including, for instance, disease-associated genetic variants [5], cancer [6], infectious diseases [7] or metabolic diseases [8]. Furthermore, the amenability of zebrafish to high-throughput experimentation enables large-scale chemical screening and toxicological studies [9,10]. However, as available screening technologies are usually tailored for cell-based assays, the execution of high-throughput and high-content screening experiments employing automated microscopy remains challenging [11,12,13]. This hampers the more widespread usage of zebrafish drug discovery pipelines in pre-clinical research settings.

The sample handling challenge generated by manipulating thousands of zebrafish specimens can usually be efficiently tackled even by smaller research laboratories. In contrast, classic zebrafish mounting procedures for microscopy are usually not compatible with large-sample volumes. Thus, zebrafish screening applications demand dedicated mounting techniques to avoid the acquisition of non-standardised image data of randomly oriented specimens [12]. Technical solutions exist and encompass sophisticated microfluidic setups [14], but also more accessible open hardware solutions for the consistent positioning of embryos within mould cavities of sample carriers compatible with widely used automated microscopes have been demonstrated [15,16,17,18].

Automated imaging and visualisation of biological events on the cell or tissue level in zebrafish is hindered by the impracticality of positioning these features precisely within the limited field-of-view (FOV) of higher magnification objectives while being mounted in microtiter plates [13,19]. Consequently, most published zebrafish screening studies rely on data acquired with low-magnification objectives with large FOVs, resulting in the reduced level of detail and informative value of phenotypic readouts. To overcome this obstacle, advanced microscopy techniques have been developed that allow for automatic detection of features of interest followed by subsequent zooming-in or other modification of the imaging workflow [19,20,21]. A drawback of these feedback microscopy methodologies is the often high level of complexity that limits their accessibility to expert personnel, who are familiar with general programming, hardware and software interfacing and advanced image processing. To enable a more widespread usage of feedback microscopy techniques by the biomedical research community and streamline advanced cell- and tissue-specific screening in whole organisms, more simplified approaches are needed.

Large-scale zebrafish screening employing automated multidimensional image acquisition leads to the generation of massive terabyte-scale image datasets [22]. Tackling the data deluge in high-content screening is a common challenge in the field of bioimage informatics, and several software packages targeted towards biomedical researchers exist [23]. Additionally, there is a large set of demonstrated analytical methods for automated image analysis for cell-based screening, and common tasks, such as cell segmentation or the extraction of morphological parameters, can often be efficiently addressed following community guidelines [24]. Unfortunately, there are no standard procedures for the analysis of zebrafish or whole organism screening data, due to the countless variety of potential phenotypic readouts at the different levels of structural organisation of the vertebrate body, its physiology and even the behaviour of the animal [22]. Therefore, zebrafish screening still mainly relies on custom-built image analysis workflows that are ideally based on common image analysis packages to ensure reproducibility and steadily expand the resource of zebrafish-specific tools.

In this study, we present an automated imaging workflow tackling every aspect of zebrafish-based screening as discussed above. This is demonstrated by the development of a high-content screening pipeline for the identification of cyst-modifying substances in a zebrafish model for human cystic kidney disease. Morpholino-based (MO) knockdown of the *intraflagellar transport 172* (*ift172*) gene was used to induce large glomerular cysts in GFP (green fluorescent protein) expressing pronephroi of the *Tg(wt1b:EGFP)* transgenic line [18,25,26]. Zebrafish embryos were treated with compounds and oriented in agarose cavities within wells of microplates generated using three-dimensional (3D)-printed orientation tools [15]. Pronephric kidneys were automatically detected in low-resolution data and subsequently imaged at higher resolution following a pre-scan/re-scan procedure, using a readily reproducible feedback microscopy workflow based entirely on Fiji [27] macros interfaced with an automated screening microscope. An analysis module for pre-processing, data filtering, automated categorisation and phenotype quantification was also realised using Fiji macros and Python scripts. The developed pipeline has been successfully applied to profile the entire Prestwick library (consisting of 1280 approved drugs) for compounds with cyst-modifying activities. Here, we present the technical implementation of the screen focussing on the engineering of image acquisition and analysis modules. This work illustrates a strategy for efficiently approaching a complex zebrafish high-content screening (HCS) project using open-source tools and software and can serve as a flexible template for organ-specific HCS assay in zebrafish, other whole organism screening or even 3D cell culture applications.

## 2. Results

### 2.1. Establishment of Disease Model and Screening Assay

The efficient utilisation of zebrafish in large-scale drug discovery applications requires robust phenotypic readouts that are compatible with automated screening assays. Zebrafish models for human cystic kidney diseases are characterised by large glomerular cysts in the developing pronephros that can be efficiently scored; however, this is often combined with other gross morphological alterations of the larval body, which can challenge the consistent data acquisition in automated imaging assays [28].

**Figure 1:**
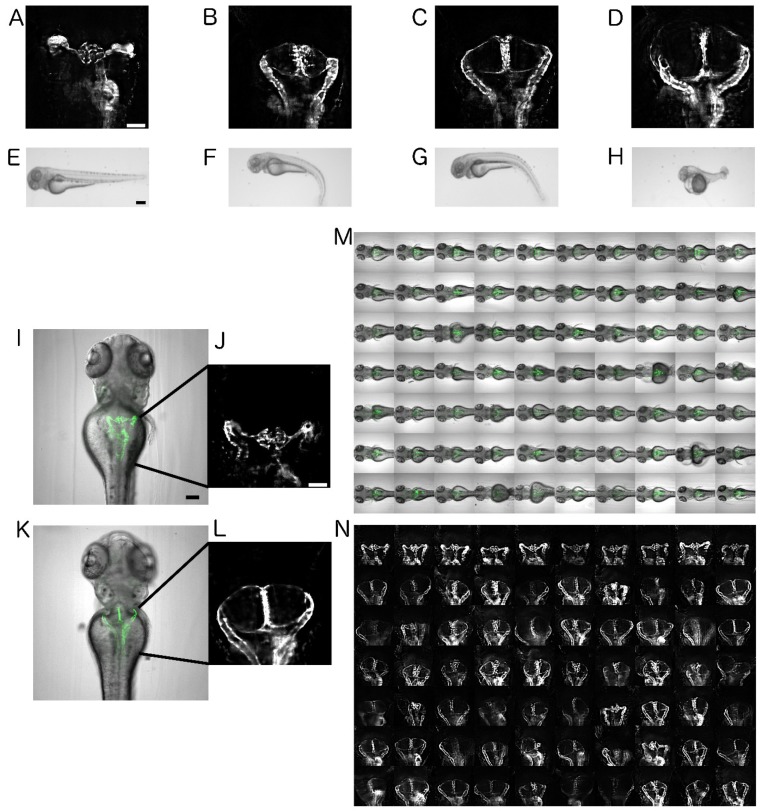
Automated imaging of an ift172-MO-based zebrafish model for human cystic kidney disease. (**A**–**D**) Dorsal views on pronephric areas in the *Tg(wt1b:EGFP)* embryos. Scale bar is 50 µm. (**E**–**H**) Bright-field stereo microscope images of the dose–response curve of ift172-MO on 72 hpf zebrafish larvae. Scale bar is 250 µm. (**A**,**E**) wild-type, (**B**,**F**) 50 µM ift172-MO, (**C**,**G**) 100 µM ift172-MO, and (**D**,**H**) 500 µM ift172-MO. (**I**,**K**) Overlay images of bright-field and fluorescence channels depicting wild-type and cystic pronephric phenotypes acquired with a 10x objective. Scale bar for I is 100 µm and J is 50 µm. (**J**,**L**) Enlarged fluorescent 10x views of I and K. (**M**,**N**) Montage images illustrating (**M**) overlay images or (**N**) pronephric areas of dorsally oriented larvae in a microtiter plate.

The intraflagellar transport protein IFT172 is involved in ciliary assembly and maintenance. Mutations in human *ift172* cause Jeune and Mainzer-Saldino syndromes, ciliopathies characterised by skeletal, renal, hepatic, or retinal abnormalities. Loss of function phenotypes in zebrafish recapitulate the severe human phenotype [25,29,30]. We exploited this to generate a zebrafish model for cystic kidney disease using splice-morpholinos-based knockdown of Ift172 in the *Tg(wt1b:EGFP)* transgenic line, resulting in readily scorable glomerular cysts (Figure 1) [18,25]. Dose–response curve experiments were carried out, to identify the morpholino concentrations that reproducibly induce glomerular cysts while keeping overall gross morphological phenotypes at an intermediate level (Figure 1A–H). While high morpholino concentrations of 100 µM and 500 µM led to gross morphological abnormalities and apparent general toxicity effects, a lower concentration of 50 µM resulted in milder overall phenotypes with reproducible formation of readily scorable glomerular cysts. Therefore, 50 µM was chosen in all the subsequent experiments. These less-impaired embryos served as a general model of renal cystogenesis, and the absence of severe malformations ensured efficient sample handling and mounting of the specimens and negligible lethality rates.

To characterise cystogenesis and identify ideal developmental windows in the search for compounds that modify cystogenesis, time-lapse experiments in Ift172-morphants were performed. Cystogenesis in morphants started around 40–44 hpf (hours post fertilization) depending on the developmental rates of the embryos (Figure S1). Around 64.3% larvae had cysts at 44 hpf, which transmuted to almost 98–100% cystic presence by 48 hpf. The cystic growth was indicated by the appearance of the lumen within the glomerular region, which was ensued by fully formed glomerular cysts observable at 72 hpf as opposed to wild-type pronephros (Figure 1I–L). At later stages, the overall morphological phenotypes of the larvae were increasingly severe without readily visible alteration of cystic kidney phenotypes (Figure S1). Thus, to avoid very early developmental toxicity effects of compounds during gastrulation and somitogenesis, the treatment period was restricted to the main cystogenesis period between 24 and 72 hpf. To enable a consistent visualisation and efficient scoring of pronephric phenotypes, we utilised a mounting strategy as previously described [15,18]. In brief, at the end of the treatment period, embryos were mounted in agarose-filled microtiter plates generated with 3D-printed orientation tools. This allowed the consistent positioning and dorsal imaging of wild-type and cystic pronephroi despite the characteristic morphological alterations of the ciliopathy phenotype such as ventrally curved tail, edemas and left-right asymmetries. (Figure 1M,N).

### 2.2. Automated Smart Microscopy of Kidney Regions

**Figure 2:**
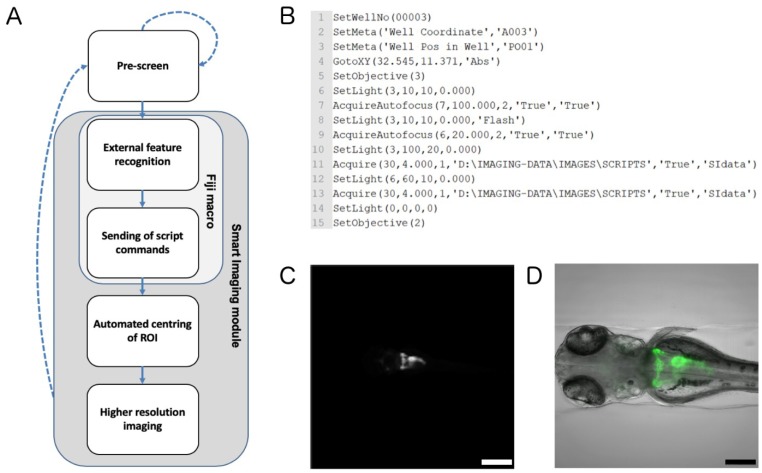
Smart imaging workflow for automated imaging of kidney regions. (**A**) Workflow chart illustrating the feedback microscopy approach utilised to automatically acquire regions of interest (ROIs). Dotted arrows indicate imaging components of the pre-screen; solid arrows indicate image processing and higher resolution imaging procedures of the smart imaging module. (**B**) Example of script commands for a single well that are sent to the automated microscope. The script triggers automated centring of the region of interest and high-resolution acquisition. (**C**) Representative overview image taken with 4x objective followed by (**D**) centring and higher resolution imaging of the pronephric region with 10x objective. The image shows a maximum projection of the GFP channel overlaid with the bright-field channel. Scale bar in C is 500µm and scale bar in D is 200 µm.

The analysis of tissue-specific phenotypes in automated zebrafish imaging assays is often limited to capturing low-resolution data with reduced informational content imposed by the usage of low-magnification objectives with large FOVs. To overcome this limitation and enable the acquisition of standardised higher resolution data of dorsal views of pronephric areas in *Tg(wt1b:EGFP)* embryos, we developed a smart imaging module following a two-step imaging procedure to automatically centre and zoom-in on regions of interest (Figure 2A). To this extent, overview data with large field of views were generated using a 4x objective and a single z-plane in the GFP channel. The directory containing acquired images was monitored by an external ‘folder-watch’ routine, which triggered the execution of a feature recognition step after the caching of new experimental images. The ‘folder-watch’ routine and feature recognition step were implemented as separate Fiji macros. The feature-recognition returns the XY-coordinates of the pronephros using centre-of-mass detection after automatic thresholding. To send the instructional feedback to the screening microscope and trigger higher resolution imaging of localised pronephric regions, the script-based smart imaging interface of the ACQUIFER Imaging Machine was used. Therefore, for each detected pronephric region a text-file containing script commands for positioning the objective lens, software auto-focus and higher resolution z-stack acquisition was generated and saved to a local directory on the control computer (Figure 2B). These ‘job-files’ were then detected and executed by the smart imaging interface of the command and control software of the microscope. As a result, each pronephric region was automatically captured at enhanced resolution in both bright-field and GFP channel using a 10x objective and 30 z-slices with a z-slice distance of 4 µm (Figure 2C,D). All the external software components of the smart imaging module were implemented in the Fiji macro language and are available in the Supplementary Information.

Using the developed approach, we acquired high-resolution image stacks of the pronephric area of more than 20,000 zebrafish embryos. Moreover, the virtually invariant location of pronephric regions and XY distribution of fluorescent signals in the acquired imaging data massively simplified many subsequent image analysis steps related to image categorisation, feature detection and phenotypic scoring. The utilised feedback microscopy approach has been entirely established using the Fiji macro language without the necessity of advanced programming environments or deep programming knowledge. The centre-of-mass-based method to locate the pronephric region is compatible with any other uniquely labelled fluorescent objects. Furthermore, it can be readily modified by combining the ‘folder watch’ macro with other image analysis routines for feature detection implemented in Fiji or other image processing tools. Ultimately, by utilising open-source image processing software coupled with a simplified feedback microscopy interface, the demonstrated imaging protocol can be easily edited and adapted by non-microscopy experts, thus enabling smart imaging applications for a wide variety of specimens.

### 2.3. Image Pre-Processing

The smart imaging screening pipeline was used to profile 1280 approved drugs for modifiers of cyst formation in the Ift172 knockdown model (Pandey [31], manuscript in preparation). To enable automatic analysis and intuitive visualisation of the generated large and complex dataset, a pre-processing protocol was developed including image restoration, automatic cropping, outlier filtering and image categorisation.

The screening system stores all the acquired images as single plane TIFF files. To facilitate data handling, all the acquired datasets were converted into multilayer TIFFs for each imaging position and channel, and widefield z-stacks were deblurred using Huygens Professional deconvolution software (as previously described in [18]). To remove any remaining out-of-focus signals and fluorescent signals originating from non-kidney structures, all the image z-stacks were reduced to seven z-slices encompassing the most focussed z-slice. Moreover, to reduce data volumes and restrict analysis to relevant information only, i.e., pronephric glomerular and tubular areas, all the image stacks were automatically XY cropped. Then, the image stacks were maximum projected, and all the subsequent analysis steps were carried out on Z-projections. This pre-processing workflow reduced the overall raw data consisting of more than 1,200,000 images (>10 Terabytes (TBs) of data) to one XYZ-cropped maximum projection for each of the approximately 20,000 embryos analysed (approximately 8 Gigabytes (GBs)). This drastically reduced data volumes, simplified file handling, enabled heuristic visualisations, e.g., plate-specific thumbnail montages (Figure 1M,N), and streamlined subsequent scoring and quantification steps. The pre-processing steps for denoising and data dimensionality reduction can be similarly applied to the processing of any other high-content screening dataset.

### 2.4. Image Quality Check and Phenotype Categorisation

**Figure 3:**
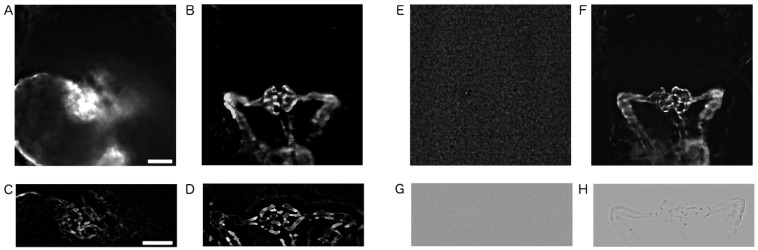
Quality control and image filtering. (**A**–**D**) Pre-processed images used for the quality check step. In both blank and blur detection process, the images acquired with 10x objective were cropped as seen in (**A**,**B**,**E**,**F**). (**C**,**D**) In blur detection, the background noise was reduced using the ‘‘Subtract Background’’ plugin in Fiji followed by variance calculation. Images below 400 variance value were classified as blurry. (**G**,**H**) In blank detection, the ‘‘Laplace Filter’’ was used before variance calculation and images above 124 variance value were considered blank. Scale bars in A and C are 50 µm.

Erroneous image data needed to be excluded from automated phenotypic scoring. Therefore, two image categories, ‘blurred’ and ‘blank’, were defined that displayed the data captured from damaged, GFP-negative, misaligned embryos or empty wells, respectively (Figure 3). These image categories were automatically identified and removed using variance calculations and edge detection algorithms (Figure 3C,D,G,H). This automated quality check removed image data from 1709 of 20,544 total wells, thus avoiding the skewing of automatically obtained quantification results caused by common mistakes occurring when manually handling and mounting of thousands of zebrafish embryos.

**Figure 4:**
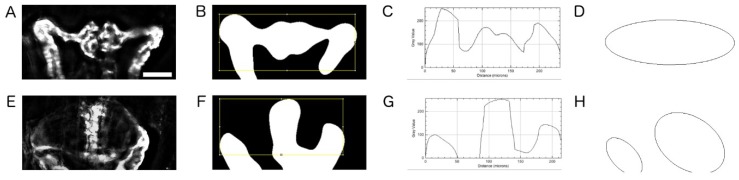
Image categorisation and phenotype detection. Pictorial representation of (**A**–**D**) wild-type and (**E**–**H**) cystic images acquired with 10x objective and checked by pronephric phenotype detection script. (**B**,**F**) Pre-processed images were denoised and thresholded. An optimised bounding box was automatically drawn to ensure the inclusion of kidney tissue. (**C**,**G**) Examples of plot profiles for control (**C**) and cystic kidneys (**G**). The difference between the resulting profiles were analysed and categorised as either wild-type or cystic kidneys. (**D**,**H**) Implementation of confirmatory script to check the previously classified pronephric phenotype. Number of ellipses surrounding the ROI and the ellipse major axis length of the largest ellipse were measured. Images with ellipse values above 299 pixels were classified as wild type. Scale bar in A is 50 µm.

In the Ift172 screen, two basic phenotypes were observed: (i) pronephros resembling the wild-type phenotype and (ii) the cystic kidney phenotype (Figure 1I,L, Figure 4). The former was observed in control morpholino (Co-MO) injected embryos, accidentally non-injected embryos and some genuine hits, while the latter was observed at different degrees of severity depending on compound treatment and experimental day for all the ift172-MO injected embryos. To perform a basic phenotypic categorisation of the entire dataset, a scoring workflow was established based on two analysis algorithms (Figure 4). First, cropped pronephros images were thresholded, and a region of interest was placed within the binary images to encompass the glomerular regions (Figure 4B,F). Then, the horizontal average intensity profile of these regions of interest in the deconvolved image was analysed. While kidneys of the wild-type phenotype display fluorescence signal across the entire region, the presence of a glomerular cyst usually leads to black image areas, visible as regions of zero intensity in image profiles (Figure 4C,G). To confirm these classification results, a secondary algorithm was used to generate a mask from binary image data showing the best fit ellipse of the detected objects. Images yielding one large ellipse were considered to be wild type, while the presence of two or multiple ellipses indicated the presence of cystic areas (Figure 4D,H). Using this image classification, wild-type and cystic kidneys could be detected with 93.7 % accuracy (N = 288).

The established workflow utilised basic image parameters to separate phenotypic categories with high accuracy, without the necessity of complex pattern recognition techniques or machine learning approaches. The strategy can also serve as a template for tasks, such as classification of complex objects, including tissue, organs or whole embryo body features, and can be readily reproduced using open-source software.

### 2.5. Quantification of Cystic Areas

**Figure 5:**
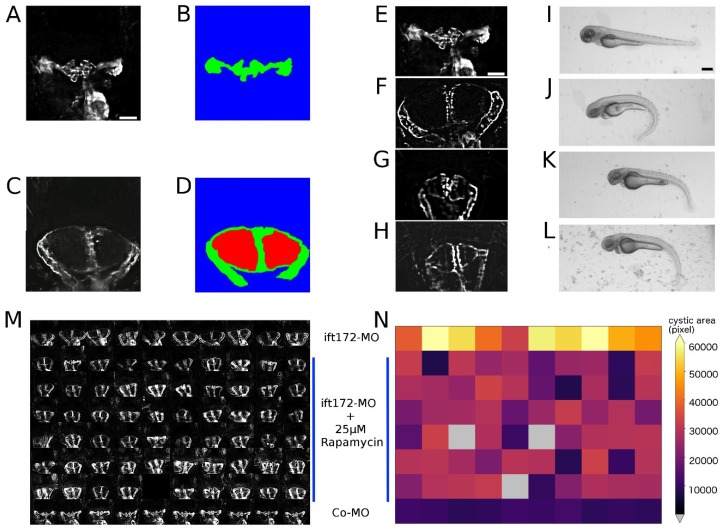
Automated scoring of pronephric phenotypes. (**A**–**D**) Quantification of pronephric tissue and cystic area in the controls (**A**,**B**) and cystic kidneys (**C**,**D**) acquired with 10x objective. Green depicts GFP-positive pronephric tissue, and red depicts cystic area. Scale bar for A–D is 50 µm. (**E**,**F**) Fluorescence and stereo microscope images of dose–response experiments administering rapamycin Co-MO or ift172-MO-injected embryos at 72 hpf. (**E**,**I**) The control, (**F**,**J**) ift172-MO injected, (**G**,**K**) ift172-MO + 25 µM rapamycin-exposed and (**H**,**L**) ift172-MO + 50 µM rapamycin-exposed larvae are shown. Scale bar for E–H is 50 µm and for I–L is 250 µm. (**M**) Montage representing 72 hpf pronephros in a microtiter plate; the first row is ift172-MO injected, last row is wild type and rest of the rows are 25 µM rapamycin-exposed larvae. (**N**) The corresponding heatmap displays rapamycin-based suppression of kidneys in reference to the cystic (first row) and wild-type (last row) controls. See also Figure S3.

To achieve rapid quantification of pronephric and cystic areas, images classified as either wild type or cystic were cropped down to their glomerular areas and binarized using global thresholding. Binary images were then analysed, and the number and morphology of foreground particles were extracted, thus providing a measure for GFP-positive areas. The particles were filtered based on position, size and morphology, and the remainder was considered to be pronephric tissue (Figure 5A,B). Then, a convex hull was constructed around foreground pixels, and the enclosed background particles were measured (https://blog.bham.ac.uk/intellimic/g-landini-software/). For images previously categorised as cystic kidneys, the background area within the convex hull was quantified as it represents the cystic area (Figure 5C,D; Figure S2). For wild-type kidney images, this background area was excluded as it represents a genuine background consisting of non-kidney tissue. Thus, the total kidney area was considered to consist of pronephric tissue for wild-type kidneys and pronephric tissue plus cystic area for cystic kidneys. The analysis strategy for measuring cyst sizes can be adapted for quantification of other features, such as lumen or other areas that are fully or partially encompassed by labelled tissue or epithelia.

The mTOR inhibitor rapamycin is known to reduce cysts in teleost and mammalian models of polycystic kidney disease, while in clinical trials in humans, it demonstrated limited efficacy as a therapeutic drug. However, to assess the efficiency of the established screening pipeline in automatically identifying reduced cyst sizes in the Ift172 model, we utilised rapamycin to carry out positive control experiments. Dose–response curve experiments were carried out, which indicated a drug concentration of 25 µM to effectively reduce but not abolish the formation of cystic areas in Ift172-morphants (Figure 5E–L). The effective concentration of rapamycin was then chosen to test all the other 1280 compounds of the Prestwick library. ift172-MO-injected embryos treated with 25 µM rapamycin were arrayed in microtiter plates, imaged using the smart imaging workflow and the image data were processed and analysed as described above (Figure 5M, Figure S3). An average of 92.1% larvae showed cystic reduction in a tested population of over 150 samples. As already shown by Tobin and Beales [32] in the *ift80* fish model, rapamycin would have probably showed its suppressive activity by repressing the hyperactivation of mTOR caused by PC1 mislocalisation. The quantified total kidney areas were plotted as heat maps allowing a rapid visualisation and identification of reduced cystogenesis (Figure 5N, Figure S3). Thus, the developed imaging and analysis workflow can be efficiently utilised to automatically identify cyst-sized modifying compounds in zebrafish high-content screening data (Supplementary protocol).

## 3. Discussion

In this work, we demonstrate the development of a zebrafish high-content screening pipeline for automated scoring of organ-specific phenotypes using intelligent screening microscopy. The pipeline was established for a chemical rescue screen in a zebrafish cystic kidney disease model and used to image over 20,000 embryos at enhanced resolution. While the biological results and associated validation studies regarding compound effects are not demonstrated in this manuscript, we highlight all the established methodological aspects that are required to conduct such a complex whole organism high-content screening assay.

For this study, a morpholino oligonucleotide-based knockdown of the *ift172* gene was utilised to mimic the human cystic kidney disease phenotype in embryos of the *Tg(wt1b:EGFP)* transgenic line [25,26]. Despite controversies regarding their usage, morpholinos remain a valuable tool to rapidly and readily alter endogenous gene expression [33,34,35]. The ift172-splice morpholino used in this study has been previously demonstrated [25], and phenocopies the *ift172* mutant background [29,30,36]. Moreover, the usage of morpholino provides significant logistical advantages when used in large-scale screening experiments, employing thousands of genetically altered embryos. Homozygous *ift172* mutants are not viable into adulthood, thus restricting husbandry to hemizygous carriers of the mutant allele. With mutants, only 25% of embryos obtained from such crosses could be used for further experimentation, thus drastically increasing animal handling and embryo usage. Additionally, mutant embryos cannot be visually phenotyped at early stages and can only be phenotyped once the pronephric cyst has formed. This would have massively reduced the efficiency of the described workflow as compounds were administered before cyst formation; therefore, using mixed populations would lead to wasting compounds and the potential masking of hits. An additional advantage of morpholino is the possibility of fine-tuning phenotype severity by using potentially hypomorphic embryos with milder gross morphological abnormalities but reproducible cyst formation. This milder phenotype allowed the usage of 3D-printed orientation tools, originally designed for 2–4 dpf wild-type embryos for reproducible mounting of specimens in agarose microtiter plates filled with moulded agarose. This ensured a standardised dorsal view on the embryonic pronephros, which was a pre-requisite to robustly score phenotypic alterations of this bilateral symmetric embryonic organ. The limitations of the chosen approach are the requirements for manual microinjection and the positioning of embryos in agarose cavities under a stereomicroscope, which can lead to a significant workload in screening scenarios. Microinjection could be potentially automated using recently demonstrated robotic injection systems, thus further increasing throughput and reducing variability [37,38]. Manual orientation of embryos takes about 15–20 min per 96-well plate; however, it is still an order of magnitude faster than traditional mounting techniques such as embedding in low melting agarose. The imaging of a full 96-well plate including pre-screen, automated centring, software autofocusing (search volume of 700 µm) and higher resolution imaging (10x NA 0.3 objective, 30 z-slices with dZ = 4 µm, 2 colour channels (NA: numerical aperture, dZ: z-slice distance)) takes approximately 40 min, so new microplates can be easily prepared during imaging times. If fewer z-slices or channels are used in different assays, imaging times would reduce drastically down to a few minutes per 96-well plate. While microfluidic methods for unsupervised automated orientation and imaging are available [14], their dependency on loading specimens into a fixed-diameter glass capillary may potentially impose challenges when malformed embryos, such as the Ift172-morphants, are used, and they seem to limit the throughput in larger scale in vivo screening experiments [39,40].

The smart imaging or feedback microscopy workflow utilised to automatically capture high-resolution datasets of pronephric regions was based on the automated detection and subsequent zooming-in on regions of interest. This overcomes the acquisition of whole embryo images at lower resolutions imposed by the random locations of regions of interest, which is a common weakness in zebrafish high-content screening assays. Besides higher magnification, the centring of the region of interest also led to very consistent and standardised image data with virtually identical locations of regions of interest within the field of views. This overall higher quality data facilitates visualisations and subsequent analysis steps. By taking advantage of a simplified hardware–software interface provided by the utilised screening microscope, the entire smart imaging workflow could be realised using the Fiji macro language without the necessity of any other programming environment or hardware communication protocols requiring engineering knowledge. Importantly, the presented smart imaging protocol can be readily modified for any other specimen by incorporating Fiji macros or scripts for the detection of other image features that are then triggered by the ‘folder watch’ macro (Supplementary Information). As Fiji is an open-source image processing tool, which is widely and massively used in the biomedical research community, we believe that the simplicity of the workflow and accessibility of the underlying tools will enable a wide range of users to conduct rather complex feedback microscopy workflows in a very similar manner.

The image analysis strategy established in this study takes advantage of the highly standardised datasets acquired. This enabled the development of a heuristic approach for massive data dimensionality reduction and outlier filtering, as well as phenotypic categorisation and scoring. The algorithms could be successfully validated by detecting altered cyst sizes upon compound treatment (Figure 5) and were used to profile a full high-content screening dataset. The developed algorithms are fully based on the open-source platforms Fiji and Python and rely on general image processing techniques and developed plugins by the open-source image analysis community. All the analyses are based on routinely used thresholding and other intensity-based techniques, edge detection algorithms and morphological operators that are commonly used in general digital image processing and that are familiar to a wide range of biomedical researchers confronted with digital imaging techniques. The algorithmic approach to tackling relatively complex, large-scale datasets can therefore be adapted for other whole organism screening studies that aim to score phenotypic alterations of embryonic organs or tissues, and certain components can be re-utilised to be incorporated in their own image analysis pipelines. The screening dataset generated using the described pipeline could potentially also be tackled using machine learning methodologies based on convolutional neural networks that are increasingly employed by the bioimage informatics community [41]. However, while these techniques seem incredibly powerful for solving complex image analysis tasks, they still require highly specialised expert knowledge and large amounts of manually annotated data to successfully train and validate established frameworks. To date, this still represents an obstacle for most biomedical research labs without access to required resources such as highly trained expert personnel.

The efficiency and simplicity of the presented methodology is illustrated by the fact that a single person was able to conduct the entire experimental work and establish the image processing pipeline, including disease model optimisation, large-scale sample manipulation and handling, automated feedback microscopy and image analysis. Once the image-based readout was established, the automated pipeline enabled the screening and profiling of a library of 1280 compounds in thousands of embryos in about one year. As the work relies on a simplified module for advanced feedback microscopy workflows and accessible open-source image processing techniques, we believe this pipeline can serve as an example and template for biomedical research labs with limited resources that aim to conduct large-scale phenotypic scoring in a whole organism model. Although, the pipeline is focussed on a drug discovery application in a pre-clinical research setting, it can be readily modified for any other large-scale imaging application in biomedical research.

## 4. Materials and Methods

### 4.1. Ethics Statement

The work presented does not involve work with animals according to German and European legislation. All experiments have been performed at stages prior to the legal onset of animal life. To obtain zebrafish embryos and larvae, fish were maintained in closed stocks at Heidelberg University. Zebrafish husbandry and experiments are under the institutional control of the Universities animal welfare agency. All the zebrafish husbandry and experimental procedures were performed in accordance with the German animal welfare standards (Tierschutzgesetz §11, Abs. 1, Nr. 1, husbandry permit number 35-9185.64/BH Wittbrodt) and in accordance with German and European Union animal welfare guidelines. The fish facility is under the supervision of the local representative of the animal welfare agency.

### 4.2. Fish Keeping and Embryo Handling

Adult zebrafish of the *Tg(wt1b:EGFP)* transgenic line [26] were kept according to the procedure described in [42]. Stage synchronised eggs were obtained via pairwise crosses in cages equipped with dividers that were removed before the eggs were required [42]. The embryos were raised at 28 °C in E3 medium.

### 4.3. Morpholino Injections

Antisense splice-blocking morpholino oligonucleotides (Gene Tools, LLC, Philomath, OR, USA) were used against the exon1-intron1 boundary of the zebrafish *ift172* gene as previously published (5’-ACGTCGTCAATATTTTACCTGAGGC-3’) [25]. Embryos at the single-cell stage were injected with 50 µM of morpholino. ift172-morpholinos resulted in similar phenotypes as published in [25,29,30]. A standard control morpholino oligonucleotide (5’- CCTCTTACCTCAGTTACAATTTATA-3’) was used as the control.

### 4.4. Drug Treatment of Embryos

At 24 h post-fertilisation, the zebrafish embryos were enzymatically dechorionated using 10 mg/mL Pronase. The embryos were transferred to a beaker, washed 3 times with 400 mL of a 1:1 solution of E3 medium/demineralised water to remove the enzymatic residues and chorions and transferred into clean petri dishes containing E3 medium [43]. Batches of 15–17 dechorionated larvae were transferred to 12-well plates containing 1.5 mL of 5 mM HEPES (Merck, Berlin, Germany)-buffered E3 medium supplemented with 0.003% 1-pheny-2-thiourea (PTU, Alfa Aesar, Karlsruhe, Germany) per well. For chemical screening, the Prestwick Chemical Library (Prestwick Chemicals, D’Illkirch, France) was used, which contains 1280 off-patent small molecules provided at 10 mM in dimethyl sulfoxide (DMSO). Larval batches were then treated with 25 µM of compounds diluted at a final concentration of 0.5% DMSO (Sigma, St. Louis, MO, USA) in E3 medium. ift172-MO-injected embryos treated with 0.5% DMSO only were used as the negative controls. The compound-containing medium was replaced every 24 h. There were a total of 1280 approved compounds in the library. At 72 hpf, the embryos were transferred into microtiter plates.

### 4.5. Preparation of Agarose-Filled 96-Well Plates and Embryo Positioning

Each well of a 96-well microtiter plate (Cat. No. 655101, Greiner, Frickenhausen, Germany) was filled with 60 µL of 1% agarose in E3 medium supplemented with 250 µg/mL tricaine (Sigma Aldrich, Taufkirchen, Germany) using a multi-channel pipette. At room temperature, the plates were inserted with a 3D-printed orientation tool to create cavities for dorsal orientation [15]. The orientation tool was carefully removed after 15 min, and the plates were wrapped in wet paper towels to be stored at 4 °C in plastic bags. At 72 hpf, the larvae were transferred in a volume of 100 µL from a 12-well plate to 96-well microtiter plates. The embryos were oriented dorsally under the stereomicroscope.

### 4.6. Automated Image Acquisition, Smart Imaging and Data Handling

96-well microtiter plates containing zebrafish embryos were automatically imaged on an ACQUIFER Imaging Machine (DITABIS AG, Pforzheim, Germany) widefield high-content screening microscope equipped with a white LED array for bright-field imaging, a LED fluorescence excitation light source, a sCMOS (2048 × 2048 pixel) camera, a stationary plate holder in combination with moving optics and a temperature-controlled incubation lid. To control the Imaging Machine, carry out image processing for feedback microscopy and data storage, an ACQUIFER HIVE was used (DITABIS AG, Pforzheim, Germany). Pronephric areas were imaged using a two-step acquisition procedure using the Imaging Machine Smart Imaging Interface (DITABIS AG, Pforzheim, Germany), i.e., kidney regions were automatically detected in lower resolution data and subsequently centred and imaged at higher resolution. In brief, the pre-scan data of GFP-labelled kidneys were acquired in the 470 nm channel using a 4x NA 0.13 objective (Nikon, Düsseldorf, Germany) and 1 z-slice. Fiji macros were developed, which monitored the directory containing the acquired images and processed each image directly after acquisition [27]. The XY-pixel coordinates of pronephric areas were detected by calculating the centre of mass after automatic thresholding and translated into XY microscope stage positions. For each XY position, the Fiji macro outputs a small script based on the ACQUIFER Script Language containing instructions for a higher resolution imaging job (Figure 2A,B; Supplementary Information). The scripts are detected by the Smart Imaging interface and automatically executed, leading to the repositioning of the objective lens to centre of the region of interest (ROI) and the execution of instructions for acquiring higher resolution data. For higher resolution imaging, data were acquired in the bright-field and 470 nm channels using 30 z-slices (dZ = 4 µm) and a 10x NA 0.3 objective (Nikon, Düsseldorf, Germany). The focal plane was detected in the 470 nm channel using a built-in software autofocus algorithm. Integration times were fixed at 60% relative LED (light-emitting diode) intensity and 10 ms exposure time for the bright-field channel and 100% relative LED intensity and 20 ms exposure time for the 470 nm channel. The acquisition of a full 96-well plate required approximately 40 min.

### 4.7. Image Pre-Processing

Raw images were sorted and converted into multilayer TIFF files of 30 slices with a custom written Perl script in combination with a Fiji macro (Supplementary Information) [18]. GFP channel multilayer TIFFs were deblurred using Huygens Professional software (Scientific Volume Imaging, Hilversum, The Netherlands) with a quick maximum likelihood estimation method and a theoretical point spread function based on microscope parameters. Batch deconvolution was performed on a workstation with 12 CPU cores/24 threads and 32 GBs of RAM. To remove out of focus slices, the image stacks were identified for the focussed slice using the “Find Focussed Slice” Fiji plugin (https://sites.google.com/site/qingzongtseng/find-focus), and a sub-stack of 7 slices was generated around it. The stacks were de-noised, maximum projected and XY-cropped by placing a bounding box of size 512 × 512 in the centre encompassing the pronephric area. These images were re-cropped by placing the bounding box in the centre with a width equivalent to the size of the image, but the height was reduced by a factor of 1.6 less than that of the original frame. This was done to include only the glomerular and pronephric tubular areas. The re-cropped images were then auto-thresholded using the Li method implemented in Fiji.

### 4.8. Image Categorisation and Image Quality Control

For each image the variance was calculated after background correction, and the images with variance values below 400 were classified as blurry and filtered out. Similarly, blank image detection was based on a variance check after Laplace filtering [44,45]. The images with variance values above 124 were considered blank and removed. To detect images displaying pronephric areas resembling the wild-type or cystic phenotypes, the re-cropped images were subjected to Gaussian blur, thresholded using Li auto-thresholding method and converted to mask in Fiji. The area fraction of the foreground pixels in these images was measured. In the images with an area fraction value of less than 100, pronephroi were selected as ROI, inverted and surrounded by bounding box. Dimensional optimisations were made to the bounding box in order to reinsure the presence of both cystic and fluorescent pronephric areas within the boundaries. On the other hand, images with area fraction values equal to 100 were first inverted and then subjected to ROI selection to exclude blank images from the evaluation scheme. The bounded areas from all the images were further plotted using the “Plot Profile” option in Fiji. The profile values were checked for the presence of zero values as an indicator of cystic presence. To confirm the wild-type versus cystic classification, “Convex Hull” was applied on the binary images followed by the “Analyze Particles” plugin to extract the lengths of the major axes of ellipses. Images with ellipse major axis lengths greater than 299 pixels were classified as wild type. Only the biggest ellipse was selected in images with more than one ellipse.

### 4.9. Image Based Quantification of Kidney Cyst Areas

To quantify the total kidney area, both cystic and pronephric areas were calculated for all the images. Pronephric areas were calculated by the “Analyze particles” function in Fiji, and areas of all the resulting particles were summed. The cystic area was analysed by inverting the binary images and drawing a convex hull around the objects. This separated the cystic area from the general background area. It was followed by the application of “Analyze particles”, leading to the formation of several bounded particles in the image, including those which represented cystic areas. All the particles were assigned numbers by the “Analyze Particles” plugin. The images here were processed so as to get the particles of interest within a count of 10. Therefore, cystic particles, among others, were selected by sequential investigation of all the particles and selecting those with a Feret diameter ≥70 and a particle number <11 per image. Areas of the selected particles were summed up to generate the final cystic area value. Finally, the images were checked for their assigned wild-type or cystic status from the classifications performed previously using the image quality control script. Based on these results, wild-type images were allocated pronephric area as the total kidney area, whereas, the cystic kidneys were attributed the sum of both pronephric and cystic areas as their total kidney area. Measurements of single microtiter plates were visualised as a heatmap using the Matplotlib library in Python.

## Supplementary Materials

Supplementary materials can be found at https://www.mdpi.com/1422-0067/20/6/1290/s1. Figures S1–S3 are available in Supplementary Figures.pdf. All scripts and macros are open-source, and are provided with a step-by-step protocol needed to reproduce the imaging workflow in Supplementary Information.zip.
