# Supplementary material for: A Smart Imaging Workflow for Organ-Specific Screening in a Cystic Kidney Zebrafish Disease Model
Source: Int J Mol Sci. 2019 Mar 14;20(6):1290. doi: 10.3390/ijms20061290 (PMC6471943; doi:10.3390/ijms20061290)
Supplement: Supplementary file 1 [file ijms-20-01290-s001.zip › J-SupplementaryInformation_revised/Source_Code/Image Analysis/PythonMethods.html]

PythonMethods


In [1]:

```
# The following script was run on folder containing multiple subfolders #
############## each containing 96 well plate data #######################
# The script goes to the folders and finds the csv files created by the #
## Fiji routine. This data is filtered and processed for final kidney ###
####### area assignment and plotting of the corresponding heatmap #######

# Importing relevant libraries #

import matplotlib.pyplot as plt
import os
import pandas as pd
import glob
import csv
import numpy as np
import re
import glob
import shutil
import subprocess
import ntpath
import re
from itertools import chain
import json
import os.path
import fnmatch
import matplotlib.pyplot as plt
import matplotlib as mpl
import plotly
```

In [2]:

```
# Entering each subfolder of the directory #

path_pattern = r'path\to\recropped\image\folder\*'
for path in glob.glob(os.path.join(path_pattern), recursive=True):
    print(path)
```

In [ ]:

```
#Pronephric areas present as separate particle values are summed together#
#and extracted from all images in the kidney folder and copied as a single# 
###### value in a new csv file corresponding to their well names ##########

writeHeader = True
All_Files = []
# checking and entering for kidney folder in each recrop subfolder #
for path in glob.glob(os.path.join(path_pattern), recursive=True):
    print(path)
    kidney_folder = glob.glob( os.path.join(path, 'Kidney\\') )  
    print (kidney_folder)
    if 1 != len(kidney_folder):
      print('SKIPPING: '+path)
      continue
    kidney_folder = kidney_folder[0]
    # Reading all files # 
    kidney_paths = glob.glob( os.path.join(kidney_folder, '*.csv') )
    writeHeader = True
    for csv_path in kidney_paths:
        df = pd.read_csv(csv_path) 
        head, tail = os.path.split(csv_path)
        df.insert(loc=0, column='Filename', value=tail, allow_duplicates=False)
        # Removing unnecessary information #
        df.drop(df.columns[[1,3,4,5,6,7,8,9,10,11,12,13,14,15,16,17,18,19,20,21,22,23,24,25,26,27,28,29]], axis=1, inplace=True)
        # Summing up the praticles to get kidney areas #
        df_grouped = df.groupby(['Filename']).agg({'Area':'sum'})
        df_grouped = df_grouped.reset_index()
        # Copying data to a new csv file #
        if writeHeader is True:
            df_grouped.to_csv(os.path.join(kidney_folder, 'kidney.csv'), mode='a', header=True, index=False)
            writeHeader = False
        else:
            df_grouped.to_csv(os.path.join(kidney_folder, 'kidney.csv'), mode='a', header=False, index=False)
```

In [6]:

```
# Copying the single csv file from kidney folder to a newly created #
######################## Analysis folder ############################

All_Files = []
for path in glob.glob(os.path.join(path_pattern), recursive=True):
    kidney_folder, = glob.glob( os.path.join(path, 'Kid*\\') ) 
    if not os.path.exists( os.path.join(path,'Analysis_folder') ):
        os.makedirs( os.path.join(path,'Analysis_folder') )
    shutil.copy(os.path.join(kidney_folder,'kidney.csv'), os.path.join(path,'Analysis_folder'))
```

In [ ]:

```
# Cystic areas present as separate particle values are filtered based on #
## certain parameters and copied as one or two values depending on the ###
###### single or double cysts detected in a new csv file corresponding ###
######################## to their well names #############################

cyst_area = []
writeHeader = True
All_Files = []
# checking and entering for kidney folder in each recrop subfolder #
for path in glob.glob(os.path.join(path_pattern), recursive=True):
    print(path)
    cyst_folder = glob.glob( os.path.join(path, 'Cyst\\') )
    if 1 != len(cyst_folder):
      print('SKIPPING: '+path)
      continue
    cyst_folder =cyst_folder[0]
    cyst_paths = glob.glob( os.path.join(cyst_folder, '*.csv') )
    writeHeader = True
    for csv_path in cyst_paths:
        df = pd.read_csv(csv_path) 
        head, tail = os.path.split(csv_path) 
        # Filtering cyst values #
        potential_cyst = df.loc[(df['MinFeret'] >= 70) & (df[' '] < 11)]
        potential_cyst.insert(loc=0, column='Filename', value=tail, allow_duplicates=False)
        # Removing unnecessary information #
        potential_cyst.drop(potential_cyst.columns[[3,4,5,6,7,8,9,10,11,12,13,14,15,16,17,18,19,20,21,22,23,24,25,27,28,29]], axis=1, inplace=True)
        # Checking for number of cysts as the script was not allowed #
        ##### to select more than two cystic particles by default ####
        total_rows = potential_cyst.shape[0]
        if((total_rows) > 2):
            potential_cyst = potential_cyst.drop(potential_cyst[potential_cyst[' '] > 11].index)
            total_rows = potential_cyst.shape[0]
        else:
            total_rows = potential_cyst.shape[0]
        if((total_rows) > 2):
            potential_cyst = potential_cyst.drop(potential_cyst[potential_cyst[' '] > 9].index)
            total_rows = potential_cyst.shape[0]
        else:
            total_rows = potential_cyst.shape[0]
        if((total_rows) > 2):
            potential_cyst = potential_cyst.drop(potential_cyst[potential_cyst[' '] > 8].index)
            total_rows = potential_cyst.shape[0]
        else:
            total_rows = potential_cyst.shape[0]
        if ((total_rows) > 2):
            potential_cyst = potential_cyst.drop(potential_cyst[potential_cyst[' '] > 7].index)
            total_rows = potential_cyst.shape[0]
        else:
            total_rows = potential_cyst.shape[0]
        if ((total_rows) > 2):
            potential_cyst = potential_cyst.drop(potential_cyst[potential_cyst[' '] > 6].index)
            total_rows = potential_cyst.shape[0]
        else:
            total_rows = potential_cyst.shape[0]
        if ((total_rows) > 2):
            potential_cyst = potential_cyst.drop(potential_cyst[potential_cyst['MinFeret'] <= 95].index)
            total_rows = potential_cyst.shape[0]
        else:
            total_rows = potential_cyst.shape[0]
        # Copying and saving data to a new csv file #
        if writeHeader is True:
            potential_cyst.to_csv(os.path.join(cyst_folder, 'cyst_area.csv'), mode='a', header=True, index=False)
            writeHeader = False
        else:
            potential_cyst.to_csv(os.path.join(cyst_folder, 'cyst_area.csv'), mode='a', header=False, index=False)
```

In [8]:

```
# Copying the single csv file from cyst folder to a newly created #
######################## Analysis folder ##########################

for path in glob.glob(os.path.join(path_pattern), recursive=True):
    cyst_folder, = glob.glob( os.path.join(path, 'Cyst\\') ) 
    shutil.copy(os.path.join(cyst_folder,'cyst_area.csv'), os.path.join(path,'Analysis_folder'))
```

In [ ]:

```
## The cystic area values are checked, if more than one value present,  ##
###### due to the presence of 2 cysts, they were merged in the same ######
###### row as 'list' to avoid muliple occurences of same filename ########

for path in glob.glob (os.path.join(path_pattern), recursive=True):
    Analysis_folder = glob.glob(os.path.join(path, 'Analysis_folder*\\'))
    if 1 != len(Analysis_folder):
        print ('SKIPPING: '+path)
        continue
    Analysis_folder = Analysis_folder[0]
    print (Analysis_folder)
    Analysis_path = glob.glob(os.path.join(Analysis_folder, 'cyst_area.csv'))
    print (Analysis_path)
    df = [pd.read_csv(cyst_area) for cyst_area in glob.glob(os.path.join(Analysis_folder, 'cyst_area.csv'))]
    df1 = pd.concat(df, axis=0)
    # Checking for multiple filename occurence and regrouping as one #
    df1 = df1.rename(columns={' ' : "Count"})
    df1.drop(df1.columns[[1,3]], axis=1, inplace=True)
    df2 = df1.groupby('Filename')['Area'].apply(list)
    # Saving as a new csv file with single filenames with mutiple area values present as 'list' #
    df2.to_csv(os.path.join(Analysis_folder, 'Cyst_Area_onerow.csv'))
```

In [ ]:

```
# The values saved as list were converted to dataframe with column names #
############ for cystic areas detected i.e. Area1 and Area2 ##############

for path in glob.glob (os.path.join(path_pattern), recursive=True):
    Analysis_folder = glob.glob(os.path.join(path, 'Analysis_folder*\\'))
    if 1 != len(Analysis_folder):
        print ('SKIPPING: '+path)
        continue
    Analysis_folder = Analysis_folder[0]
    Analysis_path = glob.glob(os.path.join(Analysis_folder, 'Cyst_Area_onerow.csv'))
    # Reading all files with cystic areas #
    df = pd.read_csv(os.path.join(Analysis_folder, 'Cyst_Area_onerow.csv'), 
                  names = ["Filename", "Area1"])
    # Checking for Area1 and Area2 and splitting them into two columns #
    df['Area2'] = df.Area1.apply(lambda x: x.split(',')[1] if len(x.split(','))>1 else '')
    df.Area1 = df.Area1.apply(lambda x: x.split(',')[0])
    df.Area1 = df.Area1.str.replace(r"\([.*]\)", "").str.extract("([0-9]+)", expand=False)
    df.Area2 = df.Area2.str.replace(r"\([.*]\)", "").str.extract("([0-9]+)", expand=False)
    df = df.fillna(0)
    #Saving as a new csv file with cystic area columns as a dataframe # 
    df.to_csv(os.path.join(Analysis_folder, 'Cyst_Area1_Area2.csv'), index=False)
```

In [ ]:

```
# Finally, each image is assigned single cystic value by summing up the #
################## cystic vaules, in case present #######################

for path in glob.glob (os.path.join(path_pattern), recursive=True):
    Analysis_folder = glob.glob(os.path.join(path, 'Analysis_folder*\\'))
    if 1 != len(Analysis_folder):
        print ('SKIPPING: '+path)
        continue
    Analysis_folder = Analysis_folder[0]
    Analysis_path = glob.glob(os.path.join(Analysis_folder, 'Cyst_Area1_Area2.csv'))
    # Reading files with all cystic areas #
    df = pd.read_csv(os.path.join(Analysis_folder, 'Cyst_Area1_Area2.csv'))
    # Summing up cystic areas and assigning them to a new column #
    df['Area_Sum'] = df['Area1'] + df['Area2']
    # Saving final cystic values as a new csv file #
    df.to_csv(os.path.join(Analysis_folder, 'Cyst_Areas_Sum.csv'), index=False)
```

In [ ]:

```
# Total kidney area is assigned here by summing up the given pronephric #
# and cystic area to each image and saving the result in a new csv file #

for path in glob.glob (os.path.join(path_pattern), recursive=True):
    Analysis_folder = glob.glob(os.path.join(path, 'Analysis_folder*\\'))
    if 1 != len(Analysis_folder):
        print ('SKIPPING: '+path)
        continue
    Analysis_folder = Analysis_folder[0]
    Analysis_path = glob.glob(os.path.join(Analysis_folder, 'kidney.csv'))
    # Reading files with summed kidney areas #
    df = pd.read_csv(os.path.join(Analysis_folder, 'kidney.csv'))
    # Reading files with summed cystic areas #
    df2 = pd.read_csv(os.path.join(Analysis_folder, 'Cyst_Areas_Sum.csv'))
    # Merging them together #
    df3 = pd.merge(df, df2, on='Filename', how='left')
    df3.fillna(0, inplace=True)
    # In the screen ctrls were always pipetted in first 12 columns # 
    ###### therefore, they were assigned only kidney areas here ####
    df3.iloc[:12, 2:5] *= 0  
    # All the areas were merged and summed in a new column #
    df3['TotalKidney_Area'] = df3['Area'] + df3['Area_Sum']
    # Saving all areas and their sum in a new csv file #
    df3.to_csv(os.path.join(Analysis_folder, 'MergedAreas_Sum_WholeKidney_Final.csv'), index=False)
```

In [ ]:

```
## Checking for the blank images in the blank folder #
######### and copying their names in a file ##########

blank_path = r'path\to\recropped\image\folder\*'
# checking and entering for kidney folder in each recrop subfolder #
for path in glob.glob (os.path.join(blank_path), recursive=True):
    Blank_folder = glob.glob(os.path.join(path, 'Blank*\\'))
    if 1 != len(Blank_folder):
        print ('SKIPPING: '+path)
        continue
    Blank_folder = Blank_folder[0]
    blank_filepath = glob.glob(os.path.join(Blank_folder, '*.csv'), recursive=True)
    last_blank_file = max(blank_filepath, key=os.path.getmtime)
    # Reading all blank folder files and scanning their variance #
    with open(last_blank_file, 'r') as f:
        words=csv.reader(f,delimiter=',')  
        merged = list(chain.from_iterable(words))
        blank_dic= dict(zip(merged[::2], merged[1::2]))
        str1 = str(blank_dic)
        blank_dic_str = eval(str1)
        with open("blank_file.csv", 'w') as outfile:
            root = csv.writer(outfile, delimiter=',')
            root.writerow(["Filename", "Variance"])
            for i,j in blank_dic_str.items():
                root.writerow([i, j])
        writeHeader = True
        blank_imgs=[]
        df = pd.read_csv("blank_file.csv")  
        df["Filename"] = df["Filename"].apply(lambda x: os.path.splitext(os.path.basename(x))[0]) 
        # Filtering files based on variance value #
        blank_img = df.loc[(df['Variance'] >= 125)]
        print (blank_img)
        # Check for the presence of null variance value #
        if blank_img.empty:
            blank_img = pd.DataFrame(index=[0,], columns=['Filename', 'Variance'])
            blank_img = blank_img.fillna(0)
            blank_img
    for b_path in glob.glob(os.path.join(Blank_folder), recursive=True):
        print(b_path)
    # Saving data to a new csv file #
    if writeHeader is True:
        blank_img.to_csv(os.path.join(b_path, 'blank_images.csv'), mode='a', header=True, index=False)       
        writeHeader = False
    else:
        blank_img.to_csv(os.path.join(b_path, 'blank_images.csv'), mode='a', header=False, index=False)
```

In [ ]:

```
# Copying the single csv file from blank folder to a newly created #
######################## Analysis folder ##########################

for path in glob.glob(os.path.join(path_pattern), recursive=True):
    blank_folder, = glob.glob( os.path.join(path, 'Blank*\\') ) 
    shutil.copy(os.path.join(blank_folder,'blank_images.csv'), os.path.join(path,'Analysis_folder'))
```

In [ ]:

```
## All blank images were assigned an outlier area value of 0 ##

for path in glob.glob (os.path.join(path_pattern), recursive=True):
    Analysis_folder = glob.glob(os.path.join(path, 'Analysis_folder*\\'))
    if 1 != len(Analysis_folder):
        print ('SKIPPING: '+path)
        continue
    Analysis_folder = Analysis_folder[0]
    Analysis_path = glob.glob(os.path.join(Analysis_folder, 'MergedAreas_Sum_WholeKidney_Final.csv'))
    # Opening files containing kidney and cystic area information #
    df = pd.read_csv(os.path.join(Analysis_folder, 'MergedAreas_Sum_WholeKidney_Final.csv'))
    df["Filename"] = df["Filename"].apply(lambda x: os.path.splitext(os.path.basename(x))[0]) 
    # Opening files containing blank image information #
    df2 = pd.read_csv(os.path.join(Analysis_folder, 'blank_images.csv'))
    # Merging the two files #
    df3 = df.merge(df2, how='left', on= 'Filename')
    df3.fillna(0, inplace=True)
    # Checking for blank images and if positive assigning them 0 values #
    df3.loc[df3.Variance != 0, 'TotalKidney_Area'] = 0
    # Saving as a new csv file with blank checked images #
    df3.to_csv(os.path.join(Analysis_folder, 'Blank_Area.csv'), index=False)
```

In [ ]:

```
## Checking for the blurry images in the blur folder #
######### and copying their names in a file ##########

blur_path = r'path\to\recropped\image\folder\*'
# checking and entering for kidney folder in each recrop subfolder #
for path in glob.glob (os.path.join(blur_path), recursive=True):
    Blur_folder = glob.glob(os.path.join(path, 'Blur*\\'))
    if 1 != len(Blur_folder):
        print ('SKIPPING: '+path)
        continue
    Blur_folder = Blur_folder[0]
    blur_filepath = glob.glob(os.path.join(Blur_folder, '*.csv'), recursive=True)
    last_blur_file = max(blur_filepath, key=os.path.getmtime)
    # Reading all blur folder files and scanning their variance #
    with open(last_blur_file, 'r') as f:
        words=csv.reader(f,delimiter=',')  
        merged = list(chain.from_iterable(words))
        blur_dic= dict(zip(merged[::2], merged[1::2]))
        str1 = str(blur_dic)
        blur_dic_str = eval(str1)
        with open("blur_file.csv", 'w') as outfile:
            root = csv.writer(outfile, delimiter=',')
            root.writerow(["Filename", "Variance"])
            for i,j in blur_dic_str.items():
                root.writerow([i, j])
        writeHeader = True
        blurry_imgs=[]
        df = pd.read_csv("blur_file.csv")  
        df["Filename"] = df["Filename"].apply(lambda x: os.path.splitext(os.path.basename(x))[0]) 
        # Filtering images based on variance value #
        blurry_img = df.loc[(df['Variance'] < 400)]
        print (blurry_img)
        # Check for the presence of null variance value #
        if blurry_img.empty:
            blurry_img = pd.DataFrame(index=[0,], columns=['Filename', 'Variance'])
            blurry_img = blurry_img.fillna(0)
            blurry_img
    for blu_path in glob.glob(os.path.join(Blur_folder), recursive=True):
        print(blu_path)
    # Saving data to a new csv file #
    if writeHeader is True:
        blurry_img.to_csv(os.path.join(blu_path, 'blurry_images.csv'), mode='a', header=True, index=False)       
        writeHeader = False
    else:
        blurry_img.to_csv(os.path.join(blu_path, 'blurry_images.csv'), mode='a', header=False, index=False)
```

In [12]:

```
# Copying the single csv file from blur folder to a newly created #
######################## Analysis folder ##########################

for path in glob.glob(os.path.join(path_pattern), recursive=True):
    blur_folder, = glob.glob( os.path.join(path, 'Blur*\\') ) 
    shutil.copy(os.path.join(blur_folder,'blurry_images.csv'), os.path.join(path,'Analysis_folder'))
```

In [ ]:

```
## All blur images were assigned an outlier area value of 0 ##

for path in glob.glob (os.path.join(path_pattern), recursive=True):
    Analysis_folder = glob.glob(os.path.join(path, 'Analysis_folder*\\'))
    if 1 != len(Analysis_folder):
        print ('SKIPPING: '+path)
        continue
    Analysis_folder = Analysis_folder[0]
    # Opening file with pre-assigned blank checked images #
    Analysis_path = glob.glob(os.path.join(Analysis_folder, 'Blank_Area.csv'))
    # Opening the file with blurry image information #
    df = pd.read_csv(os.path.join(Analysis_folder, 'Blank_Area.csv'))
    df["Filename"] = df["Filename"].apply(lambda x: os.path.splitext(os.path.basename(x))[0]) 
    df2 = pd.read_csv(os.path.join(Analysis_folder, 'blurry_images.csv'))
    # Merging the two files
    df3 = df.merge(df2, how='left', on= 'Filename')
    df3.fillna(0, inplace=True)
    # Checking for blurry images and if positive assigning them 0 values #
    df3.loc[df3.Variance_y != 0, 'TotalKidney_Area'] = 0
    # Saving as a new file with blur-blank checked images #
    df3.to_csv(os.path.join(Analysis_folder, 'Blur_Area_Final.csv'), index=False)
```

In [ ]:

```
# Filtering, detecting and copying all wildtype kidney image names to a # 
############################## new csv file #############################

writeHeader = True
wt_filenames = []
path_p = r'path\to\wild type\image\check\folder\*'
# checking and entering for wildtype check folder in each crop subfolder #
for p in glob.glob (os.path.join(path_p), recursive=True):
    wt_folder = glob.glob(os.path.join(p, 'Wt_F*\\'))
    if 1 != len(wt_folder):
        print ('SKIPPING: '+path)
        continue
    wt_filenames = []
    writeHeader = True
    wt_folder = wt_folder[0]
    # Opening each csv file attached with the image # 
    wt_paths = glob.glob(os.path.join(wt_folder, '*.csv'))
    for csv_path in wt_paths:
            print (csv_path)
            df = pd.read_csv(csv_path) 
            # Filtering files based on the presence of 0s in plot profile data #
            df.loc[df.index[0], 'Value'] = 1
            zero_csv = df.loc[(df['Value'] == 0)] 
            if zero_csv.empty:
                head, tail = os.path.split(csv_path)
                wt_filenames.append(tail)
            df1 = pd.DataFrame(wt_filenames, columns=["Filename"])
    # Saving data to a new csv file #
    if writeHeader is True:
        df1.to_csv(os.path.join(wt_folder, 'wtFilenames.csv'), mode='a', header=True, index=False)
        writeHeader = False
    else:
        df1.to_csv(os.path.join(wt_folder, 'wtFilenames.csv'), mode='a', header=False, index=False)
```

In [ ]:

```
# Copying the single csv file from wildtype check folder to a newly created #
######################## Analysis folder ##########################

path_pat = r'path\to\wildtype\image\check\folder\*'
for wt_path in glob.glob(os.path.join(path_pat), recursive=True):
    print('FROM: ' + wt_path)
    wt_folder, = glob.glob( os.path.join(wt_path, 'Wt_F\\')) 
    shutil.copy(
      os.path.join(wt_folder,'wtFilenames.csv'),
      os.path.join(wt_folder,'..','..','Recrop','Analysis_folder')
    )
None
```

In [15]:

```
# Filtering and copying all the wildtype re-check in a new csv file #

writeHeader = True
All_Files = []
path_partcle = r'path\to\wildtype\image\re-check\folder\*'
# checking and entering for wildtype recheck folder in each wttype check subfolder #
for p in glob.glob (os.path.join(path_partcle), recursive=True):
    partcle_folder = glob.glob(os.path.join(p, 'MajorCheck\\'))
    if 1 != len(partcle_folder):
        print ('SKIPPING: '+path)
        continue
    All_Files = []
    writeHeader = True
    partcle_folder = partcle_folder[0]
    # Opening each csv file attached with the image #
    partcle_paths = glob.glob(os.path.join(partcle_folder, '*.csv'))
    for csv_path in partcle_paths:
        df = pd.read_csv(csv_path) 
        head, tail = os.path.split(csv_path)
        # Selecting the highest ellipse are in case of more than one present #
        imax = df['Area'].argmax()
        df = df.iloc[imax:imax+1]
        df['Filename'] = tail
        All_Files.append(df)
        df_all = pd.concat(All_Files).set_index('Filename')
    # Saving data to a new csv file #
    if writeHeader is True:
        df_all.to_csv(os.path.join(partcle_folder, 'MajorCheck.csv'), mode='a', header=True, index=True)
        writeHeader = False
    else:
        df_all.to_csv(os.path.join(partcle_folder, 'MajorCheck.csv'), mode='a', header=False, index=True)
```

In [ ]:

```
# Copying the single csv file from wildtype re-check folder to a newly created #
######################## Analysis folder ##########################

path_pat = r'path\to\wildtype\image\re-check\folder\*'
for wt_path in glob.glob(os.path.join(path_pat), recursive=True):
    print('FROM: ' + wt_path)
    wt_folder, = glob.glob( os.path.join(wt_path, 'Major**\\')) 
    shutil.copy(
      os.path.join(wt_folder,'MajorCheck.csv'),
      os.path.join(wt_folder,'..','..','Recrop','Analysis_folder')
    )
None
```

In [ ]:

```
# Both primary and rec-checked wild type detections are assigned and the #
#################### area values are finalised here ######################

path_pattern = r'path\to\recropped\image\folder\*'
# checking and entering for analysis folder in each recrop subfolder #
for path in glob.glob (os.path.join(path_pattern), recursive=True):
    print (path)
    Analysis_folder = glob.glob(os.path.join(path, 'Analysis_folder*\\'))
    if 1 != len(Analysis_folder):
        print ('SKIPPING: '+path)
        continue
    Analysis_folder = Analysis_folder[0]
    # Opening csv file with areas assgined after blur-blank ascertainment #
    Analysis_path = glob.glob(os.path.join(Analysis_folder, 'Blur_Area_Final.csv'))
    df = pd.read_csv(os.path.join(Analysis_folder, 'Blur_Area_Final.csv'))
    # Checking wildtype check list against pre-assigned area values #
    # All wildtype detected kidneys are assigned non-cystic value here #
    df2 = pd.read_csv(os.path.join(Analysis_folder, 'wtFilenames.csv'))
    df2['Filename'] = df2['Filename'].map(lambda x: str(x)[:35])
    df2['Wt_Kidney'] = 0
    df3 = df.merge(df2, how='left', on= 'Filename')
    # Checking wildtype re-check list against pre-assigned area values #
    # In case any kidney was unassigned as wildtype it's corrected here#
    df4 = pd.read_csv(os.path.join(Analysis_folder, 'MajorCheck.csv'))
    df4['Filename'] = df4['Filename'].map(lambda x: str(x)[:35])
    df4.drop(df4.columns[[1,2,3,4,5,6,7,8,9,10,11,12,13,14,15,16,18,19,20,21,22,23,24,25,26,27,28,29,30,31,32,33,34]], axis=1, inplace=True)
    df5 = df3.merge(df4, how='left', on= 'Filename')
    df5= df5.drop_duplicates()
    after_11 = df5.index > 12
    not_nan = ~pd.isnull(df5.Wt_Kidney)
    major_check = df5['Major'] > 299
    df5.loc[after_11 & not_nan & major_check]
    kidney_area = df5['TotalKidney_Area'] != 0
    df5.loc[after_11 & not_nan & major_check & kidney_area, 'TotalKidney_Area'] = df5.Area   
    # Saving finally assgined area file as a new csv file #   
    df5.to_csv(os.path.join(Analysis_folder, 'MajorBlur_Area_wt.csv'), index=False)
```

In [ ]:

```
# Plotting heatmap based on the finally assgined areas to the images #
######### in correspondance to the microtitre plate imaged ###########

for path in glob.glob (os.path.join(path_pattern), recursive=True):
    Analysis_folder = glob.glob(os.path.join(path, 'Analysis_folder*\\'))
    if 1 != len(Analysis_folder):
        print ('SKIPPING: '+path)
        continue
    Analysis_folder = Analysis_folder[0]
    # Reading each final area file of the respective subfolder #
    df3 = pd.read_csv(os.path.join(Analysis_folder, 'MajorBlur_Area_wt.csv'),decimal=b'.')
    colormap = df3['TotalKidney_Area'].values.reshape([8,12])
    threshold = 60000 
    print (colormap)
    # Colour scheme of the heatmap #
    cmap = mpl.cm.inferno
    # Assigning plot area #
    fig = plt.figure()
    sub = fig.add_subplot(1,1,1)
    fig.suptitle('Kidney Whole Area', fontsize=16, fontweight='bold')
    norm = mpl.colors.Normalize(vmin=80, vmax=threshold) 
    cmap.set_under('silver')
    sub.set_aspect('equal')
    cb_sub=fig.add_axes([0.95, 0.1, 0.03, 0.8])
    img = sub.imshow(colormap, cmap=cmap, norm=norm, interpolation='none')
    cb = mpl.colorbar.ColorbarBase(cb_sub, cmap=cmap, norm=norm, extend='both')
    # Sets size of entire heatmap
    fig.set_size_inches(10.5,8.5, forward=True) 
    # Assigning labels and ticks to the heatmap #
    x = ['1','2','3','4','5','6','7','8','9','10','11','12']
    y = ['A','B','C','D','E','F','G','H']
    sub.set_xticklabels([]+x, minor=False)
    sub.set_yticklabels([]+y, minor=False)
    sub.set_yticks(np.arange(colormap.shape[0]), minor=False)
    sub.set_xticks(np.arange(colormap.shape[1]), minor=False)
    sub.xaxis.tick_top()
    # Heatmap plotted #
    plt.show()
    # Assigning heatmap name of the folder #
    split_path = path.split('\\')
    split_path[3]
    Heatmap_name = 'Heatmap_wt'+'_'+split_path[3]+'.png'  
    # Saving heatmap as png file #
    fig.savefig(os.path.join(Analysis_folder,Heatmap_name), format='png', dpi=600)
```
