# Supplementary material for: A Smart Imaging Workflow for Organ-Specific Screening in a Cystic Kidney Zebrafish Disease Model
Source: Int J Mol Sci. 2019 Mar 14;20(6):1290. doi: 10.3390/ijms20061290 (PMC6471943; doi:10.3390/ijms20061290)
Supplement: Supplementary file 1 [file ijms-20-01290-s001.zip › J-SupplementaryInformation_revised/I - Supplementary Figures_revised.pdf]

Supplementary Materials: A smart-imaging workflow for organ-specific screening in a cystic kidney zebrafish disease model

Gunjan Pandey, Jens H. Westhoff, Franz Schaefer, Jochen Gehrig

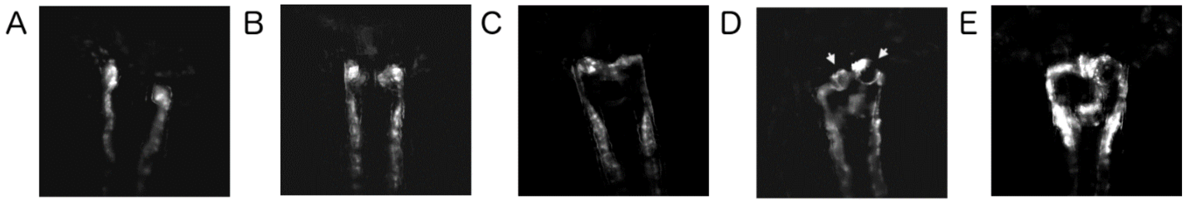

Figure S1. Developmental analysis of cyst formation in ift172 morphants. Time lapse recording performed at (A) 24 hpf, (B) 32 hpf, (C) 40 hpf, (D) 44 hpf, arrows indicate forming cysts (E) 48 hpf to analyse the cystogenesis timeline of ift172 MO injected *Tg(wt1b:EGFP)* zebrafish embryos. 44 hpf was seen as the critical time point for cyst development in more than 60% larvae.

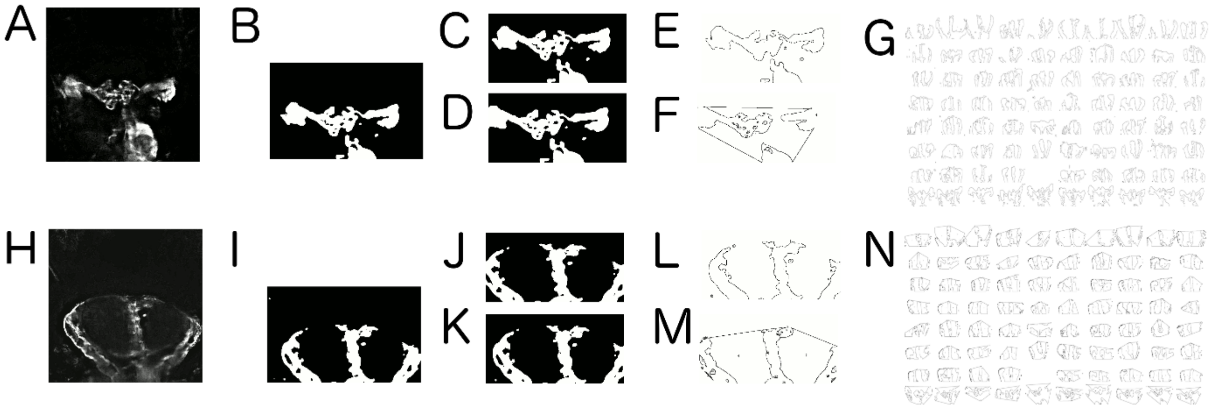

Figure S2. Automated measurement of pronephric and cystic areas. (A-G) and (H-N) are the pictorial representation of wild type and cystic area quantification method, respectively. (A,H) show the initial XYZ-projected image used by the script, which are further cropped and thresholded (B,I). This is followed by (C,D) and (J,K) where the images are recropped to focus precisely on the glomerular region. In (E-M) the images are binarised and are subjected to ''Analyze Particles'' plugin both before (E,L) and after (L,M) drawing convex hull around ROI. (E,L) present the pronephric area whereas, (F,M) pictorially depict the cystic area acquisition. All areas once collected are filtered through selective parameters and assigned to images based on their pre-defined status (i.e. wild type or cystic) decided earlier by the quality control step. (G,N) are the corresponding montages showing the collated binary images used for the pronephric and cystic area calculation respectively.

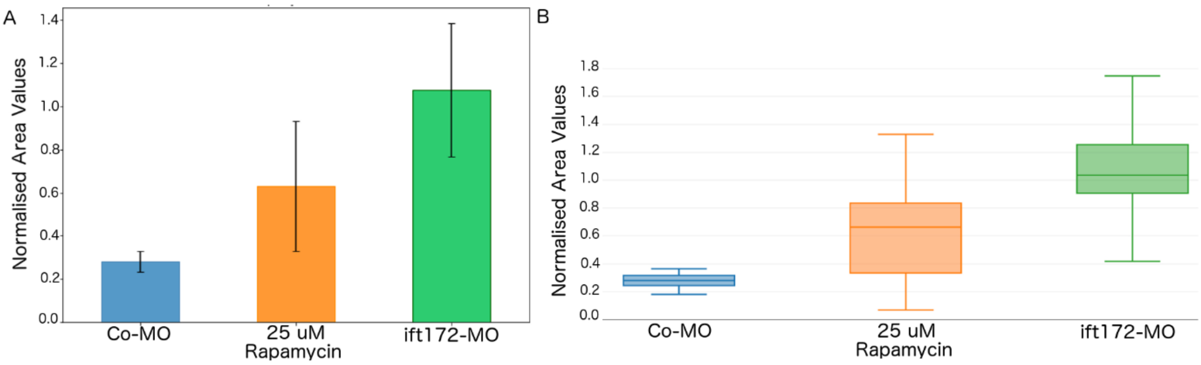

Figure S3. Quantitative analysis of cyst sizes upon rapamycin treatment. (A) Bar chart showing the area quantification results of Co-MO (blue; mean=0.27, SD=0.04) , ift172-MO + 25 uM rapamycin (orange; mean= 0.63, SD=0.3) and ift172-MO injected larvae (green; mean=1.07 , SD=0.3) at 72 hpf. The plots represent the normalised data against the plate-specific mean of negative controls (i.e. ift172-MO injected larvae). (B) Box plots indicating the distribution of individual area measurements. Whiskers indicate min-max values.
