# Supplementary material for: A Smart Imaging Workflow for Organ-Specific Screening in a Cystic Kidney Zebrafish Disease Model
Source: Int J Mol Sci. 2019 Mar 14;20(6):1290. doi: 10.3390/ijms20061290 (PMC6471943; doi:10.3390/ijms20061290)
Supplement: Supplementary file 1 [file ijms-20-01290-s001.zip › J-SupplementaryInformation_revised/Supplementary protocol.pdf]

Step-by-step protocol for execution of image processing tools for automated quantification of cyst areas

Supplmentary information for:
A smart-imaging workflow for organ-specific screening in a cystic kidney zebrafish disease model

Gunjan Pandey[1,2], Jens H. Westhoff[1], Franz Schaefer[1], and Jochen Gehrig[2]

[1]Department of Pediatrics I, University Children's Hospital Heidelberg, Heidelberg, Germany
[2]ACQUIFER is a division of Ditabis AG, Pforzheim, Germany

For questions related to this protocol or the associated modules, please contact: gunjan.pandey@med.uni-heidelberg.de

Procedure

Download all the script files from the supplementary folder. If you wish to make no changes to the script and execute the scripts in their given state, please install all the dependencies mentioned at the end of the document.

A Automated image acquisition script

1. Download: Supplementary folder >> Smart Imaging sub-folder >> "SmartImaging_macro.ijm" script.

2. Launch the control software of the ACQUIFER Imaging Machine along with the smart imaging (SI) interface.

3. Configure the required imaging parameters for the pre-screen on the Graphical User Interface (GUI), such as, integration time, objective, channels etc.

4. Launch the Fiji software and install the "SmartImaging_macro.ijm" as plugin under Fiji >> Plugins >> Install.

5. Start the pre-screen experiment on the ACQUIFER Imaging Machine by clicking on "Generate Script" and then 'Start'.

6. As the imaging commences, go to the "Jobs Folder (..\JOBS) " in the file explorer and wait for the file "Image_Location.pth" to appear and then, execute the pre-installed SI macro in the Fiji plugins section to begin automated feature detection and acquisition of region of interest (ROI).

   Note: SI script parameters can be easily modified to fit the demands of the project, for example: cells, tissues, organs etc. could be captured.

7. All images will be saved in the pre-defined project folder.

B Image pre-processing module

B.1 Automated multi-layer generation

1. Download: Supplementary folder >> Multilayer Generation sub-folder >> "MultilayerTiff-Script.pl".
2. Pool all the imaged folders requiring multi-layer generation in a single folder.
3. Launch Fiji and the multilayer generating Perl script.

   Note: The Perl script can be modified, for example: channels subjected to multilayer tiff generation can be decided from the list of acquired channels, folder location change etc.

4. Copy the address of the target folder in the Perl window >> press Enter.
5. Go to the target folder >> find "makemulti-macro.ijm" >> drag and drop to Fiji window >> run script.
6. All folders will end up with a new "multitiff" folder with sub-folders for all imaged channels and multilayer tiff files for the channel subjected to the script.

B.2 Image restoration

1. Launch Huygens 3D deconvolution software.
2. Open a single image >> parameter window >> find theoretical PSF >> enter other required settings >> save the settings file for batch deconvolution later.
3. Open batch processing mode >> select target folder >> create save folder >> execute pre-generated with defined parameters as deconvolution script.
4. All deconvolved images will end in the pre-defined save folder, ready for further processing.

C Automated quantification script

If you choose to make no changes to the script:

1. Download: Supplementary folder >> Image Analysis sub-folder >> "AllFijiScripts.ijm".
2. Launch Fiji >> Open scripts >> Run "Script1" >> Select folder.
3. Script will create the specified folder sub-folders >> Crop >> Recrop >> 1) Cyst, 2) Kidney and 3) Analysis Folder
4. Upon "Script1" execution, the "Crop" folder contains cropped and z-projected versions of the images that were sub-stacked around their focal plan using "Find Focussed Slide" plugin.

   Note: All parameters involved in this sub-module can be changed as per project requirements.

For example: focal plane settings, z-projection settings, number of slides used for sub-stacking, ROI selection etc.

5. The "Recrop" folder contains recropped, thresholded and outlier filtered images from the "Crop" folder.

   Note: All parameters involved in this sub-module can be changed as per project requirements. For example: chosen threshold, denoising threshold, ROI selection etc.

6. "Cyst" folder contains the results of cystic area calculation in ".csv" format for all the images from "Recrop" folder.

7. "Kidney" folder contains the results of kidney area calculation in ".csv" format for all the images from "Recrop" folder.

   Note: The location of convex hull drawn is ROI dependent and can be modified as per ROI in question, along with other parameters in ***Steps 6 and** 7*.

8. Open "Blank Detection" script >> Run script.

9. The script will create a sub-folder in the "Recrop" folder and save the variance results in ".csv" format for all the images from the "Crop" folder.

10. Open "Blur Detection" script >> Run script.

11. The script will create a sub-folder in the "Recrop" folder and save the variance results after "Subtract background" plugin execution in ".csv" format for all the images from the "Crop" folder.

12. Open "Wild type Detection" script >> Run script.

13. The script will create a sub-folder in the "Crop" folder and save the "Plot profile" results in ".csv" format for all the images from the "Crop" folder.

14. Open "Wild type/Cyst Detection re-check" script >> Run script.

15. The script will create a sub-folder in the wild type detection folder and save the "Analyze particles" >> Ellipse measurement results in ".csv" format for all the images from the "Crop" folder.

16. For the upcoming steps, Python 3.0 was used in " Jupyter notebook" environment on Windows 10 platform. The script can also be run on a Mac System with some modifications.

    Note: The script has the capacity to be executed in other Python environments, but we have not tried that in our project.

17. Launch the "Jupyter notebook" using Windows 10 command prompt and execute the python script >> "PythonMethods.ipynb". The script will stepwise go through all the results folders with ".csv" files.

18. The areas from individual "Kidney" files will be added and collated together in a single file

against the file name. This collated file with kidney areas for all imaged wells is copied to the "Analysis Folder".

19. The individual "Cyst" files will be filtered to screen for particles with desired cystic morphology and collated and saved in a new file against the file name as its cystic area. The collated file with cyst areas for all imaged wells are copied to the "Analysis Folder".

20. The individual "Blank" and "Blur" files will be screened and the well names clearing set threshold limit will be collated. These lists with the filenames will be copied to the "Analysis Folder".

21. The individual files from wild type detection folders will be checked and the well names clearing the set "Plot profile" parameters will be collated. These lists with the filenames will be copied to the "Analysis Folder".

22. The results from "Kidney", 'Cyst", "Blank", "Blur", "wild type" lists for each file well will be merged in a single ".csv" file.

    Note: All the threshold values are subject to the ROI in question and can be changed depending on the required end-point in ***Steps 17–21***.

23. The python script will go through each column in a row i.e. Blank, Blur, wild type and check for the assigned status. Based on which, the final kidney value will assigned to that well. For instance: a wild type kidney will be assigned only pronephric area value, whereas cystic kidney will be assigned pronephric area + cystic area values. On the contrary, blank and blur images will be assigned zero-area values, in order to prevent data skewing due to false positives.

24. These assigned area values will be further plotted as heatmap corresponding to the 96-well plate format imaged using 'Matplotlib' library. The heatmap labelled by the name of the folder analysed will be saved in the "Analysis Folder" location.

Note: The location of the folders can be changed to any preferred location.

Dependencies required for the script execution:

Certain plugins which are not part of the default Fiji library are used and must be installed in advance for the script execution:

– "Find Focussed Slice" (Link: https://sites.google.com/site/qingzongtseng/find-focus)

– "Convex Hull Plus" (Link: https://blog.bham.ac.uk/intellimic/g-landini-software/)
– "Laplace Filter" (Link: http://mosaic.mpi-cbg.de/?q=downloads/imageJ)

– Python 3.0 was used in "Jupyter notebook" environment on Windows 10 platform.

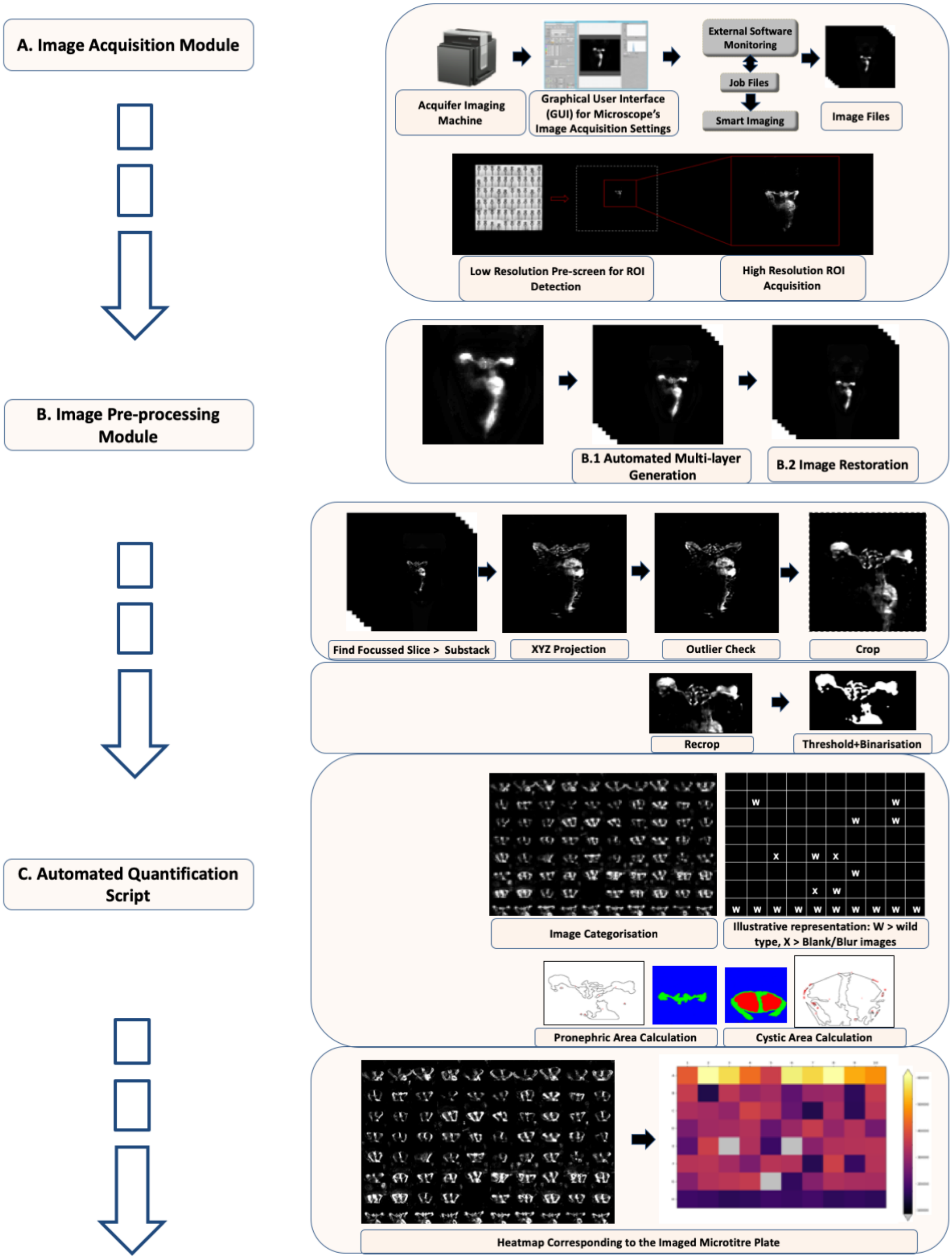

Figure: Pictorial illustration of step-by-step protocol for execution of image processing tools for automated quantification of cyst areas
